# Incarcerated hernia after internal hemipelvectomy for G2 chondrosarcoma: a preventable complication?

**DOI:** 10.1111/ans.15148

**Published:** 2019-03-04

**Authors:** Andrea Baltic, Christian Laback, Thomas Auer, Andreas Leithner, Gerwin A. Bernhardt

**Affiliations:** ^1^ Department of Orthopaedics and Trauma Medical University of Graz Graz Austria; ^2^ Division of Plastic, Aesthetic and Reconstructive Surgery Department of Surgery, Medical University of Graz Graz Austria; ^3^ Division of General Surgery, Department of Surgery Medical University of Graz Graz Austria

**Figure 1:**
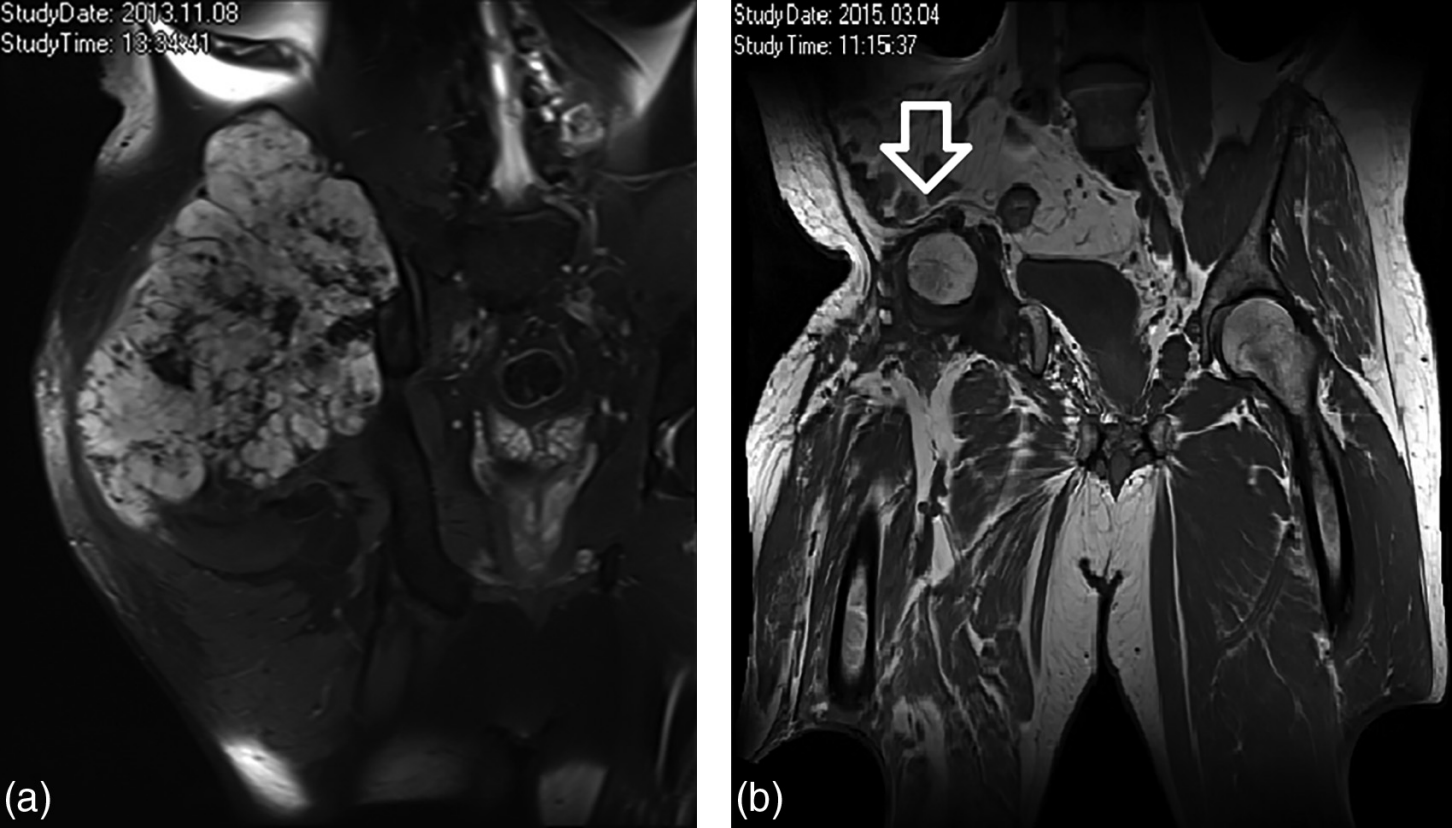
(a) Chondrosarcoma infiltrating and destructing the ilium (22 × 16 × 19 cm). (b) No sign of hernia or tumour recurrence (arrow pointing at intraperitoneally onlay mesh (IPOM) position).

A 53‐year‐old severely obese patient (body mass index 36.3) with a visible, hard, non‐mobile mass in the right gluteal region presented to our clinic. The mass was known for 20 years and had been previously diagnosed as an osteochondroma. Magnetic resonance imaging (MRI), however, showed an infiltrating tumour causing destruction of the ilium, as can be seen in Figure 1a. An incisional biopsy of the mass revealed a G2 chondrosarcoma.

We performed an internal hemipelvectomy with R0 margins, combined Type I and II pelvic resection according to Enneking, without hip joint reconstruction. The patient was discharged 14 days after surgery.

Six weeks after discharge, he developed acute abdominal pain. A computed tomography (CT) scan revealed an incarcerated hernia. This was treated by open surgery with a pelvic incision, reduction of the herniated bowel and inferior onlay mesh closure.

Soon after this intervention, the patient developed peritonitis and re‐incarceration. He was transferred to our clinic with sepsis just 2 months after the initial surgery. The short time frame here suggests the possibility of a surgical site infection at the time of mesh insertion or, although unlikely, a missed perforation at the time of hemipelvectomy as potential cause for the further complications. Median laparotomy was performed immediately and the infected mesh was removed. Localized bowel perforation necessitated an ileocoecal resection. The abdomen was left open with an abdominal negative pressure system *in situ.*

**Figure 2:**
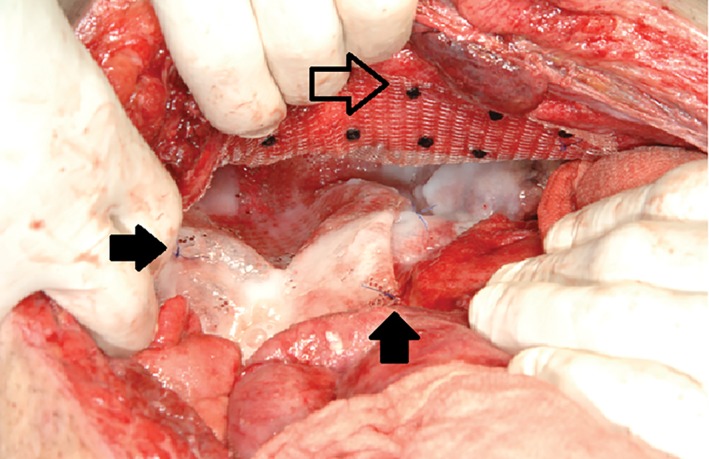
Intraperitoneally onlay mesh (IPOM) fixed with resorbable tacks (outlined arrow), fibrin glue and resorbable sutures (full arrow).

After two abdominal dressing changes the hernia gap was closed using an intraperitoneal onlay mesh (IPOM) fixed with resorbable tacks laterally. Medially, fibrin glue and resorbable sutures were used, as tacks would have affected vessels and nerves (Fig. 2). The large pelvic skin defect was reconstructed using a modified latissimus dorsi flap.

After a total stay of 40 days, the patient was fit for discharge.

As part of our routine follow‐up protocol for chondrosarcoma, the patient underwent local MRI of the pelvis and radiological staging with chest CT scans and abdominal sonography every 3 months for the first 3 years after surgery and biannually thereafter.

More than 4 years later, the patient has no sign of hernia recurrence and has had an uneventful oncological follow‐up (Fig. 1b).

Since chemotherapy and radiation therapy are not effective in the context of chondrosarcomas, wide resection with clear margins is the only treatment option.^1^ Internal hemipelvectomy always leads to a weakness of the abdominal wall, including loss of pelvic floor integrity. These results, per definition, in a hernia gap.^2^ Consequently, 100% of cases result in hernias. Whereas most patients remain asymptomatic, symptomatic hernias may lead to a life‐threatening situation, which as this case suggests, could be prevented.^3^

Besides a weakened abdominal wall after radical resection, other factors including genetic aspects and increased abdominal pressure can lead to pelvic incarceration through the surgically produced hernia gap.^4^, ^5^ In our case, the patient's overweight was the most important risk factor known to increase the recurrence rate.^6^ In these patients, prophylactic mesh reinforcement may avoid such complications. Onlay mesh fixation from the pelvic site however should be avoided because of difficult fixation options and lack of peritoneal stabilization as seen in our case.^7^ Moreover, onlay techniques show higher recurrence rates than sublay procedures.^8^ An IPOM technique after incarceration initially, may have resulted in more favourable outcomes. Other potential solutions could have been an initial laparoscopic IPOM repair simultaneously at the time of radical tumour resection, or prior to tumour resection in a separate operation.

Due to the lack of published literature there are no guidelines supporting such a procedure or even establishing when the optimal time for such a prophylactic hernia surgery would be.

It has been recently shown, that the use of a prophylactic IPOM may significantly reduce hernia development in incisional hernias.^8^ Similarly, good results after hemipelvectomy with additional IPOM procedure could be expected.

As long as there is no evidence supporting either approach, cases like this require an interdisciplinary work‐up with orthopaedic, general, plastic and reconstructive surgical teams to discuss possible strategies adapted to individual patients' needs. Such potentially fatal cases like ours could possibly be avoided with the adequate surgical procedure at the time of first operation or at least after developing complications.

Further investigations are necessary to identify criteria for selecting patients who would benefit from a prophylactic mesh reinforcement. We therefore propose that patients should be entered into a prospective database to better analyse complications such as symptomatic hernias after hemipelvectomy.
